# Supplementation of mitochondria from endometrial mesenchymal stem cells improves oocyte quality in aged mice

**DOI:** 10.1111/cpr.13372

**Published:** 2022-12-08

**Authors:** Qi Zhang, Jian‐Xiu Hao, Bo‐Wen Liu, Ying‐Chun Ouyang, Jia‐Ni Guo, Ming‐Zhe Dong, Zhen‐Bo Wang, Fei Gao, Yuan‐Qing Yao

**Affiliations:** ^1^ Medical School of Chinese People's Liberation Army General Hospital Beijing China; ^2^ Department of Obstetrics and Gynecology The First Medical Center of Chinese PLA General Hospital Beijing China; ^3^ State Key Laboratory of Stem Cell and Reproductive Biology Institute of Zoology, Chinese Academy of Sciences Beijing China; ^4^ Department of Clinical Biobank Center The Medical Innovation Research Division of Chinese PLA General Hospital Beijing China; ^5^ University of Chinese Academy of Sciences Beijing China; ^6^ Shenzhen Key Laboratory of Fertility Regulation The University of Hong Kong‐Shenzhen Hospital Shenzhen China

## Abstract

Maternal ageing is one of the major causes of reduced ovarian reserve and low oocyte quality in elderly women. Decreased oocyte quality is the main cause of age‐related infertility. Mitochondria are multifunctional energy stations that determine the oocyte quality. The mitochondria in aged oocytes display functional impairments with mtDNA damage, which leads to reduced competence and developmental potential of oocytes. To improve oocyte quality, mitochondrial supplementation is carried out as a potential therapeutic approach. However, the selection of suitable cells as the source of mitochondria remains controversial. We cultivated endometrial mesenchymal stem cells (EnMSCs) from aged mice and extracted mitochondria from EnMSCs. To improve the quality of oocytes, GV oocytes were supplemented with mitochondria via microinjection. And MII oocytes from aged mice were fertilized by intracytoplasmic sperm injection (ICSI), combining EnMSCs' mitochondrial microinjection. In this study, we found that the mitochondria derived from EnMSCs could significantly improve the quality of aged oocytes. Supplementation with EnMSC mitochondria significantly increased the blastocyst ratio of MII oocytes from aged mice after ICSI. We also found that the birth rate of mitochondria‐injected ageing oocytes was significantly increased after embryo transplantation. Our study demonstrates that supplementation with EnMSC‐derived mitochondria can improve the quality of oocytes and promote embryo development in ageing mice, which might provide a prospective strategy for clinical treatment.

## Graphical abstract

In this study, we chose endometrial mesenchymal stem cells (EnMSCs) as the sources of mitochondria. We isolated the EnMSCs from 10‐month‐old mice and then extracted the mitochondria of EnMSCs. Then, the GV oocytes and MII oocytes from aged mice were injected with mitochondria. We found that mitochondria derived from EnMSCs could significantly improve the quality of oocytes, promote the embryonic development and improve the birth rates of aged mice.

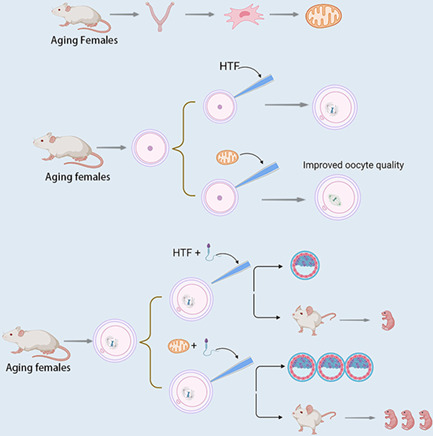

## 1 INTRODUCTION

Maternal ageing is becoming an increasingly relevant issue in many countries.^1^ Female fertility tends to decline with age, with features including reduced ovarian reserve, decreased oocyte quality and reproductive endocrine changes, which ultimately result in decreased pregnancy rates, decreased live birth rates and increased miscarriage rates.^2^ Decreased quality of oocytes is considered to be the vital contributor to age‐related declines in fertility, but the problem of maternal age can be effectively abrogated by oocyte donation. For elder women, it has been reported that the defects in meiotic and cytoplasmic ability are associated with oocyte ageing.^3^, ^4^ After 35 years, female fertility declines significantly, and approximately 50%–70% of mature oocytes from 40‐year‐old women have chromosomal abnormalities.^5^ Additionally, mitochondrial dysfunction is one of the most notable cytoplasmic changes in aged oocytes and causes decreased cytoplasmic competence.^6^

For oocytes, mitochondria are the multifunctional powerhouses and provide energy to support oocyte maturation, including spindle formation and chromosome segregation.^7^ It is widely accepted that paternal mitochondrial DNA (mtDNA) is actively eliminated during early embryogenesis and that the quality of mitochondria in oocytes is essential for embryo development.^8^, ^9^ Mitochondrial DNA contains 37 genes, of which 22 are for transfer RNA 2 are for the large and small subunits of ribosomal RNA and 13 are for proteins (polypeptides). Oocytes have the most mitochondria among all cell types, and the number of mitochondria can expand from 6000 per primordial oocyte to 40,000 or more per mature oocyte at the end of metaphase II (MII).^10^

Mitochondrial dysfunction accumulates with advancing maternal age. Mitochondrial dysfunction can include quantitative damage, such as a decreased mtDNA copy number, or qualitative damage, such as mtDNA strand breaks, point mutations and oxidative damage. Several factors can contribute to mitochondrial dysfunction in oocytes. Since mtDNA is near the electron transport chain and reactive oxygen species (ROS), it may be more subject to mutational burden than nuclear DNA. However, the detailed molecular mechanisms remain to be explored.^11^ Mitochondrial dysfunction can lead to oocyte maturation arrest, defective spindle formation and abnormal chromosome segregation, ultimately resulting in embryonic chromosomal aneuploidy.^12^, ^13^ In recent years, mitochondrial supplementation has been proposed as a strategy to improve the quality of aged oocytes. There are some kinds of cells that are used as sources of mitochondrial supplementation, such as oogonial germline stem cells (OSCs), liver cells, granulosa cells and cumulus cells. However, the existence of OSCs is still controversial, and the extraction process is complex.^14^, ^15^ Supplementation with mitochondria from liver cells does not improve embryo development, and granulosa or cumulus cells are also affected by maternal ageing.^16^, ^17^ Furthermore, the process of mitochondrial extraction from the above cells is invasive.^18^ Therefore, choosing a suitable source of mitochondria is challenging for mitochondrial supplementation therapy.

Studies in the past decade have shown that mitochondria from mesenchymal stem cells (MSCs) can regenerate and repair damaged cells or tissues.^19^ Endometrial mesenchymal stem cells (EnMSCs) are located in the endometrium and can be extracted from menstrual blood and used to treat diseases such as intrauterine adhesions.^20^ In this study, we demonstrated that purified EnMSCs can be stably cultured with relatively superior mitochondrial function compared to that of primary endometrial stromal cells (ESCs) in a mouse model. We also found that the transfer of mitochondria from EnMSCs can improve oocyte quality and fertility in aged mice. Importantly, these findings might provide a prospective strategy for clinical treatment, during which the entire procedure for EnMSC collection would be noninvasive.

## 2 METHODS AND MATERIALS

### 2.1 Animals

All mouse experiments were performed in accordance with the principles approved by the Institutional Animal Care and Use Committee of the Institute of Zoology, Chinese Academy of Sciences (CAS; SYXK 2021‐0063). Eight‐month‐old ICR mice were acquired from SPF Beijing Vital River Laboratory Animal Technology Co., Ltd., and then were further raised to 10 months of age for use in the experiments.

### 2.2 Harvest and culture of EnMSCs and primary ESCs

EnMSCs and primary ESCs were isolated from 10‐month‐old mice. First, the uterus was dissected from the fallopian tube on both sides and washed with phosphate‐buffered saline (PBS), and then the adipose tissue and other tissues connected to the uterus were removed. Second, the uterus was cut into several pieces and digested in PBS containing 2 mg/ml collagenase I (Worthington, LS004196). The tissue fragments were digested at 37°C for 60 min at 180 rpm. Third, after digestion, the samples were filtered through a 70‐μm filter and then centrifuged at 1600 rpm for 8 min. Finally, Complete MesenCult™ Expansion Medium (Stem Cell, 05513) and DMEM/F12 medium with 10% FBS, 100 U/ml penicillin and 100 μg/ml streptomycin were used to cultivate EnMSCs and primary ESCs at 37°C in a humidified environment with 5% CO_2_.

### 2.3 Cell proliferation assay of EnMSCs

The proliferation of cells was evaluated using MTT assays.^21^ Briefly, cells were seeded into 24‐well plates at a density of 2 × 10^3^ cells per well for 7 days, and the detection lasted 7 days. At each detection time point, the cells were cultured in an incubator with 500 μl of MTT solution (0.5 mg/ml) for 4 h at 37°C. After removing the MTT solution, the cells were incubated with 750 μl of DMSO on a shaking table at 75 rpm for 10 min. Then, the OD values at 490 nm were evaluated by a microplate reader (BioTek PowerWave XRS). The proliferation curve of EnMSCs was drawn with time (days) on the horizontal axis and OD values on the vertical axis by using GraphPad Prism version 9.0.0.

### 2.4 Flow cytometry

To identify characteristic markers expressed on EnMSCs, the cells were separated with 0.25% trypsin–EDTA and incubated with antibodies against CD34 (eBioscience, 11034181), CD45 (eBioscience, 11045181), CD29 (Invitrogen, 12029181), CD73 (eBioscience, 12073181) and CD90 (eBioscience, 11090081) for 40 min. After being washed three times with PBS and centrifugation, the cells were resuspended in 300 μl of PBS and analysed by flow cytometry (BD Fortessa). The obtained data were analysed by FlowJo software. The expression of different markers was analysed by FlowJo software after removing cellular debris and adherents by drawing gates.

For detection of mitochondrial membrane potential, the mitochondrial probe JC‐1 was used according to the manufacturer's instructions (Beyotime, C2006). The cells were stained with 0.02 mg/ml JC‐1 for 20 min at 37°C. Then, flow cytometry analysis (BD Fortessa) was used to detect the mitochondrial membrane potential (△Ψ*m*) after washing and resuspending the cells in 400 μl of PBS. The mean fluorescence intensity of JC‐1 monomers (green fluorescence) and JC‐1 aggregates (red fluorescence) was analysed by FlowJo software after removing cellular debris and adherents by drawing gates. The relative red/green fluorescence intensity was used to evaluate ΔΨm in mitochondria.

For intracellular ROS measurement, a ROS assay kit was used according to the instructions (Beyotime, S0033S). Then, 10 μM DCFH‐DA was used to incubate the cells at 37°C for 20 min. Following washing and centrifugation, the cells were resuspended in PBS, and flow cytometry (BD Fortessa) was used to analyse fluorescence. After removing cellular debris and adherents by drawing gates, the mean fluorescence intensity of DCFH‐DA was analysed by FlowJo software.

### 2.5 Osteogenic and adipogenic differentiation

Osteogenic and adipogenic differentiation of MSCs was performed with kits according to the instructions (Cyagen, MUXMX‐90021, MUXMX‐90031). For osteogenic differentiation, cells were plated into six‐well plates and cultured in complete medium until they reached 70% confluency. Then, the complete medium was replaced with a differentiation medium, and culture continued for 2 weeks. Alizarin Red staining was used to identify osteogenic differentiation; osteoblasts were stained red by the Alizarin Red. Adipogenic staining when the cells reached approximately 100% confluence. Then, the medium was replaced with differentiation medium, and culture continued for 3 weeks. Then, the cells were stained with Oil Red O to identify adipogenic differentiation. The adipocytes were stained red with Oil Red O under a microscope. For both experiments, the differentiation media were changed twice per week, and images were captured by an inverted microscope (Nikon, Eclipse Ti‐s).

### 2.6 Mitochondria extraction, microinjection and intracytoplasmic sperm injection

Mitochondria extraction was performed according to a recently reported protocol.^22^ Briefly, the cells were resuspended in human tubal fluid (HTF, Millipore, MR‐070‐D) at a concentration of 10^6^ per ml and homogenized well. Then, the samples were centrifuged twice, and the pellets were resuspended in 1 μl of HTF. Mitochondria (7–8 pl) in HTFs were microinjected into each GV oocyte. The microinjection process was completed within 30 min via a Nikon operating system. For MII oocytes, 10 pl of mitochondria combined with intracytoplasmic sperm injection (ICSI) were microinjected into each oocyte. ICSI was performed as described previously.^23^ Sperm heads were moved into mitochondria‐HTF drops, and then a processed sperm head was injected into a MII‐stage oocyte via an inverted microscope. After microinjection, the oocytes were washed in KSOM (Sigma, MR‐107‐D) and cultured at 37°C in a CO_2_ incubator.

### 2.7 Detection of mtDNA copy numbers by quantitative real‐time PCR

Quantitative real‐time PCR to assess oocyte mtDNA was conducted as previously described.^24^ The mouse mtDNA‐specific primers B6 forward, AACCTGGCACTGAGTCACCA, and B6 reverse, GGGTCTGAGTGTATATATCATGAAGAGAAT, were used to prepare external standards for absolute quantification of mtDNA. To obtain the standard curve, PCR products were ligated into a T‐vector, and the standard curve was generated with seven 10‐fold serial dilutions of purified plasmid standard DNA. Briefly, one oocyte was loaded in a PCR tube with 10 μl of lysis buffer and incubated at 55°C for 2 h. After incubation at 95°C for 10 min to heat‐inactivate Proteinase K, the samples were used directly for qPCR analysis. An ABI system and mouse mtDNA‐specific primers were used to conduct qPCR. Linear regression analysis of all standard curves for samples showed a correlation coefficient higher than 0.98. All measurements were performed in triplicate.

### 2.8 Oocyte collection and embryo transfer

Ten‐month‐old mice were used to obtain GV‐ and MII‐stage oocytes. At 48 h after the injection of PMSG (Ningbo, China) into oocytes, GV‐stage oocytes were retrieved from the ovaries of mice and moved to M2 medium (Sigma, m7167) containing 0.2 mM IBMX (Sigma, i5879). After microinjection, the oocytes were washed and moved into IVM medium containing TCM‐199 (Gibco, 12340030) supplemented with 10% FBS (Gibco, 10099141C), 0.2 mmol/L sodium pyruvate (Sigma, P5280), 2 mmol/L L‐glutamine (Stem Cell, 07100), 10 IU/ml PMSG (Ningbo, China), 10 IU/ml HCG (Ningbo, China), 1 μg/ml β‐oestradiol (Sigma, E2758) and 10 ng/ml EGF for culture in a 37°C incubator containing 5% CO_2_. For MII oocytes, mice were injected with HCG 48 h after PMSG, MII oocytes were retrieved from fallopian tubes 12 h after HCG (Ningbo, China) injection, and cumulus cells were digested by hyaluronidase (1 mg/mL, Sigma, H3506). The fertilized oocytes were transferred to the oviducts of surrogate mothers on an ICR background.

### 2.9 Immunofluorescence staining

For primary ESC validation by vimentin/cytokeratin staining, the cells were fixed with 4% paraformaldehyde (PFA) for 30 min and permeabilized with 0.2% Triton X‐100 for 15 min. After blocking with 5% bovine serum albumin (BSA) for 1 h, the samples were incubated with primary antibodies overnight at 4°C and then with secondary antibodies for 1 h at room temperature after washing with PBS. Then, 4′,6‐diamidino‐2‐phenylindole (DAPI, 1:1000, Thermo Fisher Scientific, S36938) was used to stain the samples after three washes, and images were captured with a Nikon DS‐Ri1 CCD camera. The primary and secondary antibodies are as follows: anti‐vimentin (1:250, Abcam, ab92547), cytokeratin (1:400, Abcam, ab7753), Cy3‐conjugated donkey anti‐rabbit IgG (1:300, Jackson ImmunoResearch, 711‐165‐152) and TRITC‐conjugated donkey anti‐mouse IgG (1:150, Jackson ImmunoResearch, 715‐025‐150).

To detect the mitochondrial membrane potential (△Ψ*m*) of oocytes after injection, the oocytes were incubated with JC‐1 working solution for 20 min at 37°C. Then, the oocytes were observed by confocal microscopy after washing in M2 medium. Images were captured with a confocal laser scanning microscope (Zeiss LSM 880). The mean fluorescence intensity of JC‐1 monomers (green fluorescence) and JC‐1 aggregates (red fluorescence) was analysed by ImageJ software (NIH, Bethesda, MD). The relative red/green fluorescence intensity was used to evaluate ΔΨm in mitochondria.

For spindle and chromosome staining, the steps before blocking were approximately the same as those for vimentin staining. After blocking with 5% BSA, the cells were incubated with a fluorescein isothiocyanate‐conjugated a‐tubulin antibody (1:600) for 2 h at room temperature. The oocytes were washed three times and stained with DAPI. A confocal scanning microscope (Zeiss LSM 880) was used to examine the samples. Then, spindle morphology and arrangement of chromosomes were assessed in both groups.

Chromosome spreads were created as previously described.^25^ Briefly, the oocytes were moved into Tyrode's solution (Sigma, T1788) to remove the zona pellucida. The oocytes were then moved onto slides containing a drop of chromosome spread solution. After drying and blocking with 1% BSA, the samples were incubated with primary antibodies overnight at 4°C and then with secondary antibodies for 1 h at room temperature. After three washes, the slides were stained with DAPI and then captured with a confocal scanning microscope (Zeiss LSM 880). The primary and secondary antibodies were as follows: anti‐centromere (1:50, Antibodies Incorporated, 15‐234) and Cy™5‐conjugated donkey anti‐human IgG (1:200, Jackson ImmunoResearch, 709‐175‐149).

### 2.10 Statistical analysis

Experiments were biologically repeated three times, and the data are presented as the mean ± SEM. ImageJ software (NIH, Bethesda, MD) was used to analyse the fluorescence intensity. To ensure the reliability of the fluorescence intensity statistics, the images for the two groups were obtained with the same settings by the confocal microscope. For quantitative statistics, an unpaired‐samples *t*‐test was carried out by using GraphPad Prism version 9.0.0, and *p* < 0.05 was considered to indicate significance.

## 3 RESULTS

### 3.1 In vitro culture and characterization of mouse EnMSCs

**FIGURE 1:**
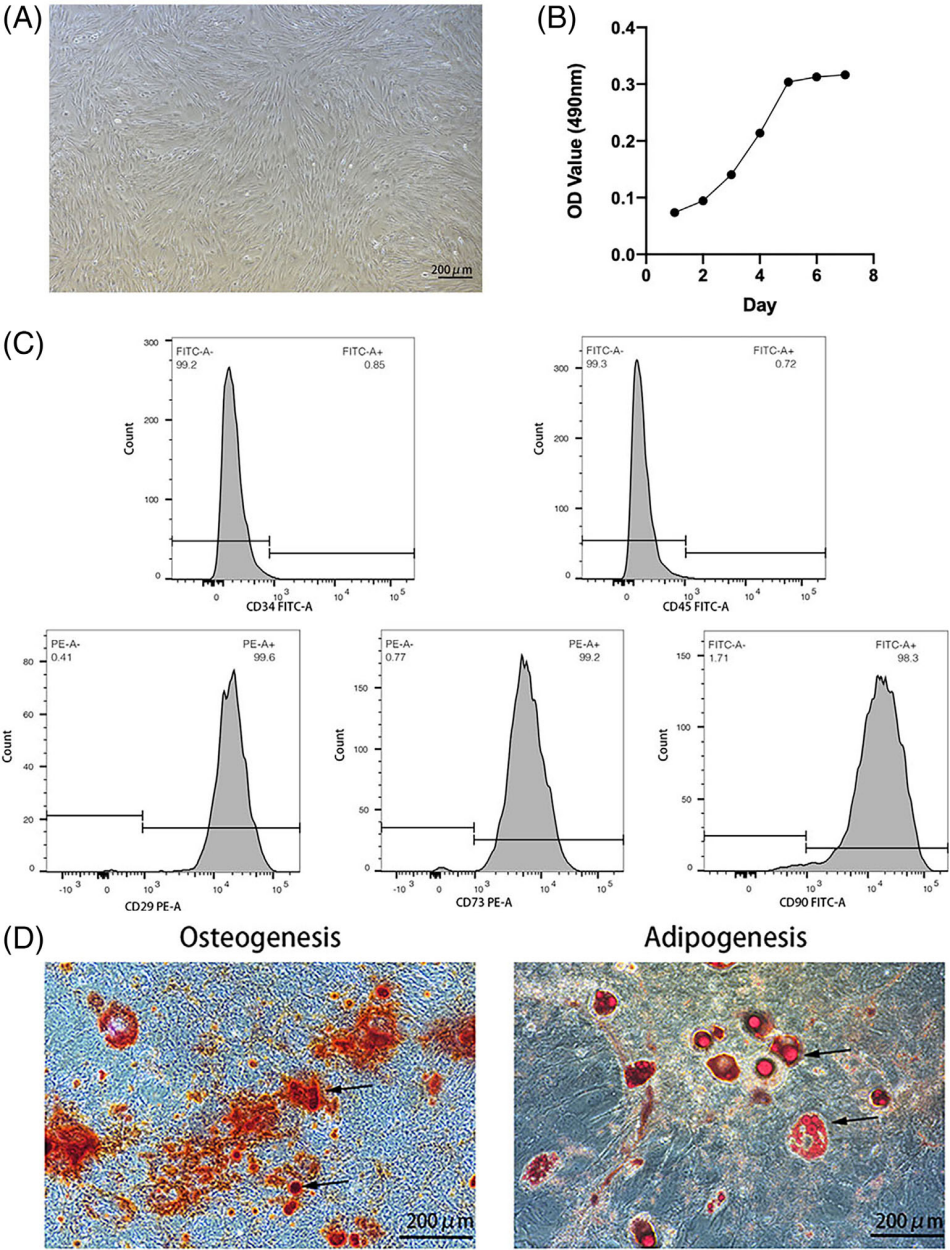
Harvest and identification of endometrial mesenchymal stem cells (EnMSCs). (A) EnMSCs displayed a typical fibroblast morphology. Bar = 100 μm. (B) EnMSCs exhibited a well‐proportioned cell growth curve. (C) Flow cytometry analysis of EnMSCs. The cells were negative for CD34 (0.85%) and CD45 (0.72%) and positive for CD29 (99.6%), CD73 (99.2%) and CD90 (98.3%). (D) EnMSCs differentiated into osteoblasts and adipocytes. The arrows indicate osteoblasts and adipocytes, respectively.

EnMSCs were isolated from 10‐month‐old mice and cultured in vitro (Figure 1A,B). The identity of derived EnMSCs was verified by flow cytometry using specific cell surface markers. We found that cultured EnMSCs were positive for CD29 (99.6%), CD73 (99.2%) and CD90 (98.3%) but did not express CD34 (0.85%) or CD45 (0.72%), which fulfiled the International Society for Cellular Therapy criteria^26^ (Figure 1C). We also found that the derived EnMSCs could differentiate into osteoblasts and adipocytes under in vitro conditions (Figure 1D). These results indicated that we successfully derived MSCs from mouse endometria.

### 3.2 Superior mitochondrial membrane potential and reduced ROS levels in EnMSCs

**FIGURE 2:**
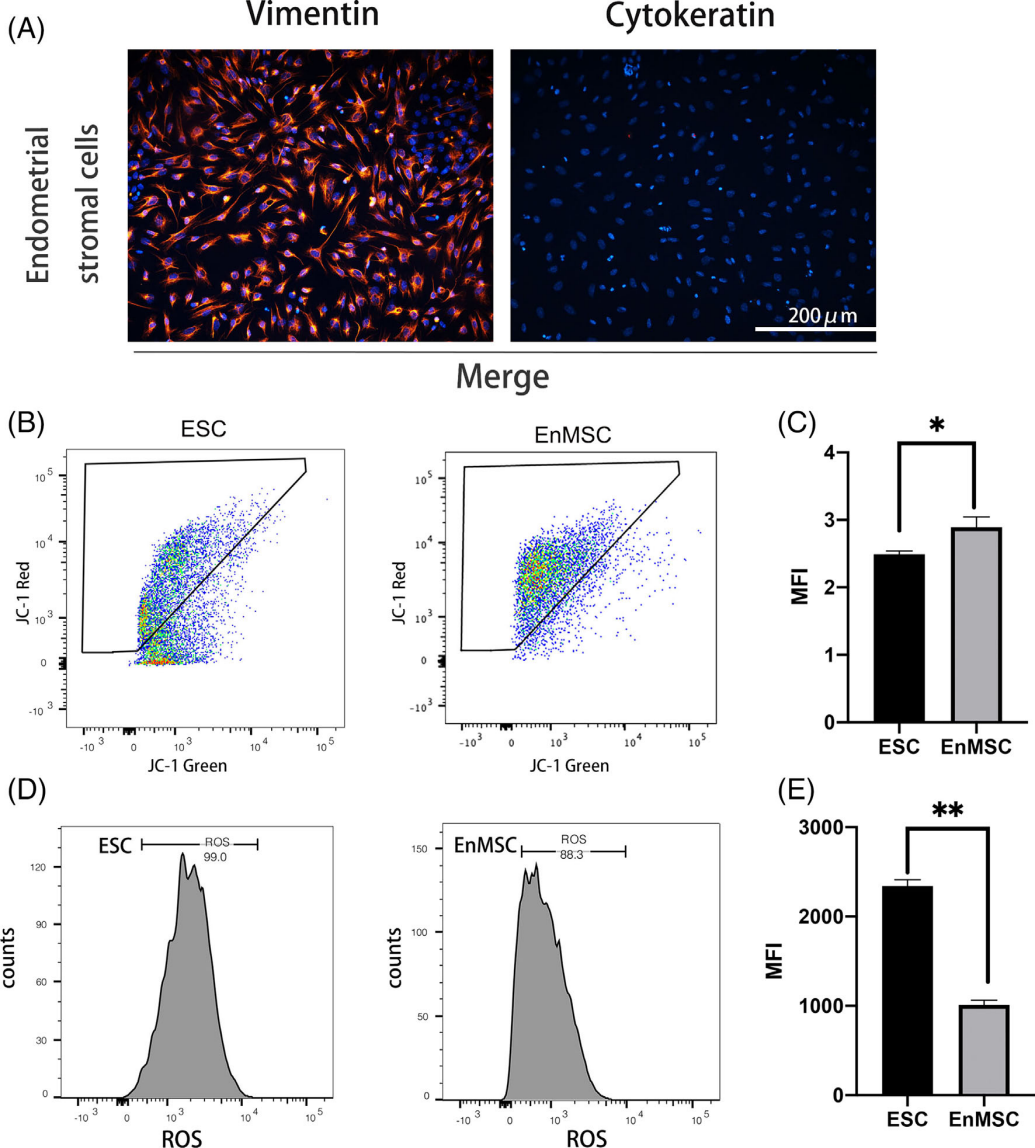
Endometrial mesenchymal stem cells (EnMSCs) displayed higher mitochondrial membrane potential (ΔΨm) and lower reactive oxygen species (ROS) than primary endometrial stromal cells (ESCs). (A) Identification of ESCs by immunofluorescence staining. Primary ESCs were positive for vimentin expression and negative for cytokeratin expression. Bar = 200 μm. (B) Flow cytometry analysis was carried out to detect the △Ψm in primary ESCs and EnMSCs by JC‐1 staining. (C) Red/green fluorescence intensity was calculated in both kinds of cells, and EnMSCs had a higher △Ψm than primary ESCs. (D) ROS levels were measured by DCFH‐DA fluorescence in both cell lines via flow cytometry. (E) A reduced ROS fluorescence intensity was found in primary ESCs. In (C) and (E), the data are the mean ± SEM of three replicates. One asterisk (*) indicates statistically significant differences at *p* < 0.05, and two asterisks (**) indicate statically significant differences at *p* < 0.01.

To examine the mitochondrial function of EnMSCs isolated from aged mice, we chose primary ESCs as the controls. Primary ESCs were isolated from aged mice and characterized by immunofluorescence analysis. Primary ESCs expressed the classic fibroblast cell marker vimentin and were negative for cytokeratin (Figures 2A and S1). Mitochondrial membrane potential (△Ψm) and the intracellular ROS levels of the ESCs and EnMSCs were measured by flow cytometry to evaluate mitochondrial function. The ΔΨm was assessed by JC‐1 staining. JC‐1 forms a polymer and produces red fluorescence when ΔΨm is high, and JC‐1 produces green fluorescence as a monomer when ΔΨm decreases. The relative red/green fluorescence intensity was used to evaluate ΔΨm in mitochondria. We found that ΔΨm in EnMSCs was much higher than that in primary ESCs (2.49 ± 0.03 vs. 2.89 ± 0.09, *p* < 0.05) (Figure 2B,C). ROS are produced by oxidative stress, cause DNA damage and promote apoptosis. DCFH‐DA is an ideal fluorescent probe widely used to detect ROS levels. We found that the fluorescence intensity of DCFH‐DA in EnMSCs was significantly lower than that in primary ESCs (2341 ± 41.14 vs. 1010 ± 30.18, *p* < 0.01) (Figure 2D,E).

### 3.3 EnMSC mitochondria improved the maturation rates and quality of oocytes from aged mice

**FIGURE 3:**
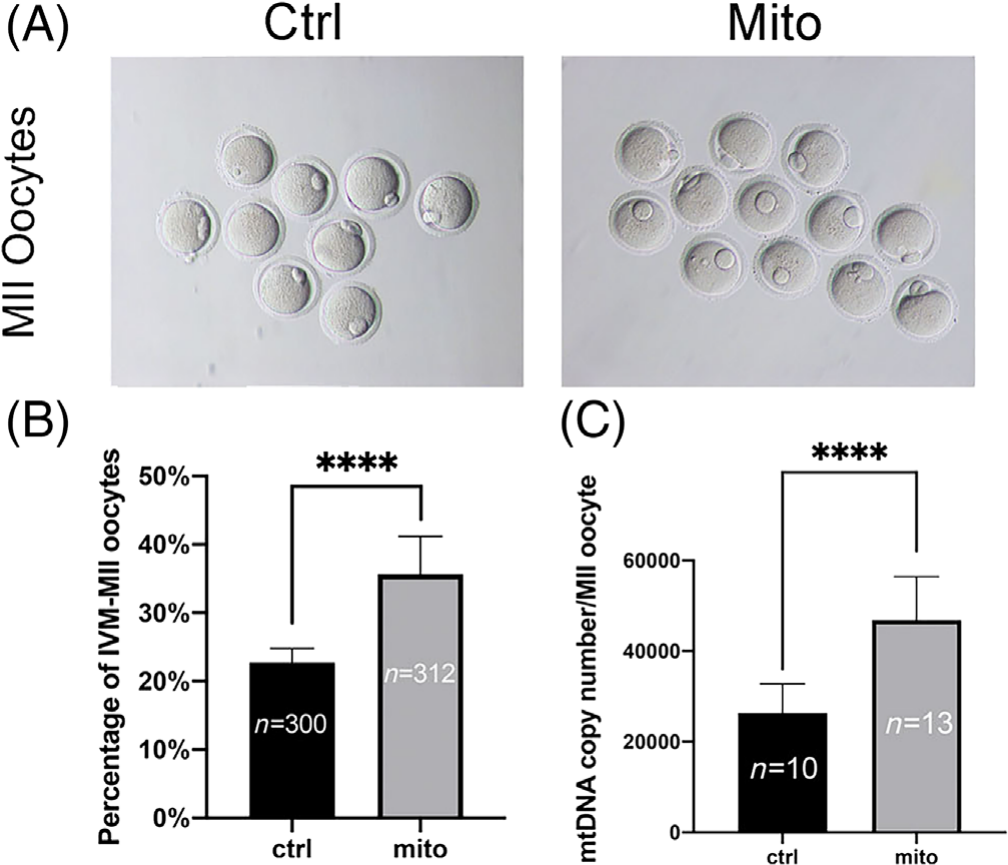
Increased maturation rates and mtDNA copy numbers in oocytes of aged mice after endometrial mesenchymal stem cell (EnMSC) mitochondrial transfer. GV oocytes from aged mice were injected with EnMSC mitochondria in human tubal fluid (HTF) in the experimental group (mito‐group) and with HTF in the control group (ctrl‐group). (A) Typical images of MII oocytes in the two groups after microinjection. Bar = 200 μm. (B) The group microinjected with mitochondria had higher maturation rates of MII oocytes than the control group. (C) The mtDNA copy numbers of oocytes in the mito‐group increased significantly after EnMSC mitochondrial supplementation compared to the numbers in the control group. In (B) and (C), the data are the mean ± SEM; *n* shows the number of oocytes, and asterisks (****) indicate statistically significant differences (*p* < 0.0001).

To examine the effects of EnMSC mitochondria on the quality of aged oocytes, GV oocytes from aged mice were injected with EnMSC mitochondria in HTF, and control aged oocytes were injected with HTF. After in vitro maturation, the percentage of oocytes at the MII stage (22.75% ± 0.67% vs. 35.65% ± 1.75%, *p* < 0.0001) (Figure 3B) and the mtDNA copy number (2.6 × 10^4^ ± 2.0 × 10^3^ vs. 4.7 × 10^4^ ± 2.7 × 10^3^, *p* < 0.0001) (Figure 3C) were significantly increased in aged oocytes with mitochondrial supplementation. These results indicated that mitochondrial injection could promote oocyte maturation.

**FIGURE 4:**
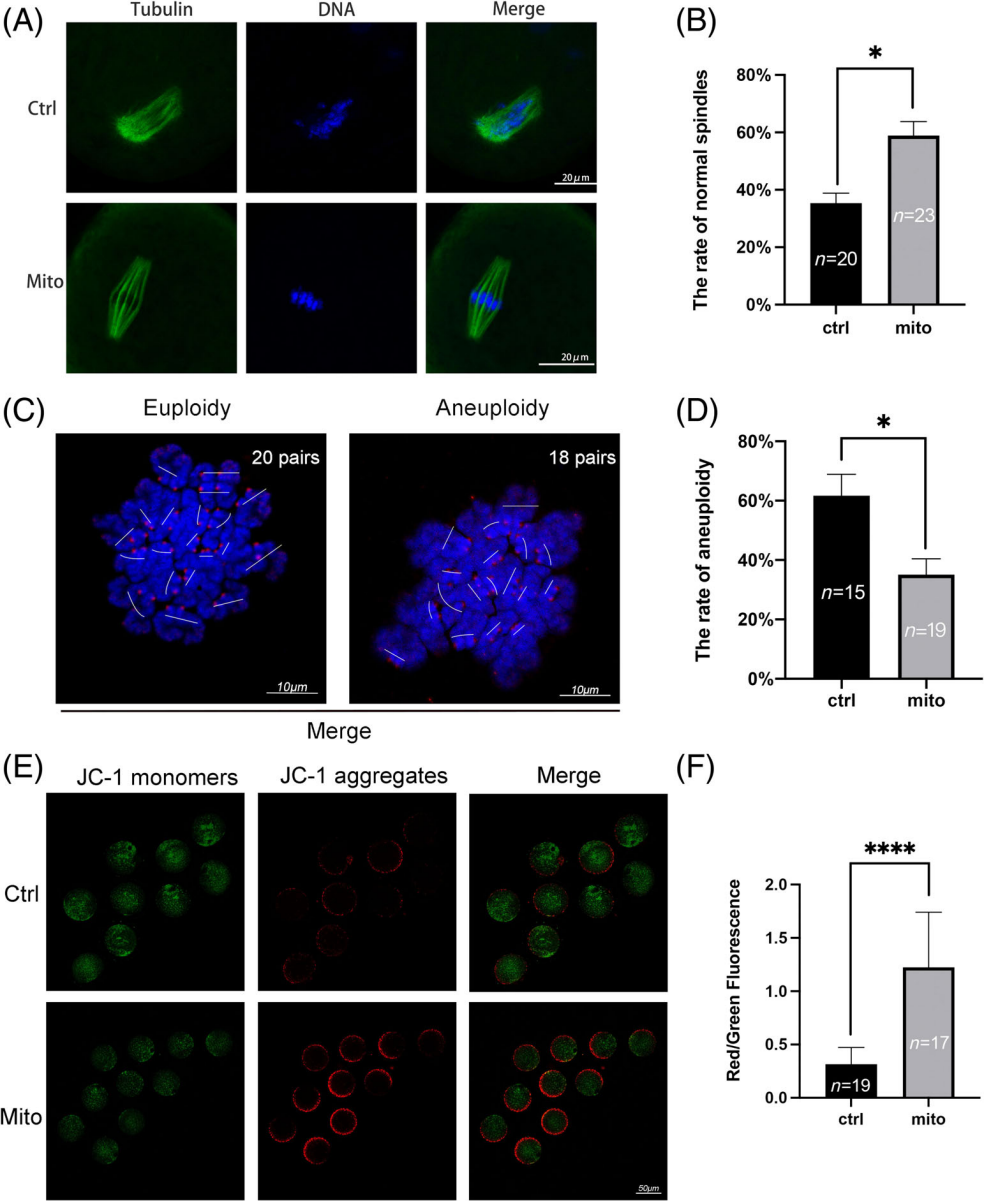
The quality of oocytes from aged mice was improved by endometrial mesenchymal stem cell (EnMSC) mitochondrial transfer. (A) Representative pictures of the spindles and chromosomes in the two groups. (B) Increased normal spindle rates were observed in MII oocytes from aged mice after mitochondrial supplementation. Bar = 20 μm. (C) Images of chromosome spreads are shown. Bar = 10 μm. (D) Decreased aneuploidy rates were found in MII oocytes after mitochondrial transfer. (E) The mitochondrial membrane potential (△Ψm) values of the two groups were assessed by JC‐1 staining in MII oocytes after mitochondrial supplementation. JC‐1 monomers with green fluorescence revealed a low △Ψm, while JC‐1 aggregates with red fluorescence revealed a high △Ψm. Bar = 50 μm. (F) Increased △Ψm was observed in MII oocytes from aged mice after mitochondrial supplementation. The data are the mean ± SEM. In C, D and F, the numbers of oocytes are denoted by *n*. One asterisk (*) represents statistically significant differences at *p* < 0.05, and four asterisks (****) represent statistically significant differences at *p* < 0.0001.

Abnormal spindle assembly and defects in meiosis are also features of aged oocytes. Hence, the spindle morphology and karyotype of aged oocytes were examined by confocal scanning microscopy. As shown in Figure 4, disintegrated spindle poles, irregularly arranged chromosomes and high rates of aneuploidy were observed in control oocytes. The spindle morphology was significantly improved (35.37% ± 3.46% vs. 58.89% ± 4.84%, *p* < 0.05) (Figure 4A,B), and the rate of aneuploidy was significantly reduced (61.67.89% ± 7.27% vs. 35.12% ± 5.29%, *p* < 0.05) (Figure 4C,D) in oocytes injected with mitochondria. Furthermore, we evaluated the mitochondrial membrane potential (△Ψm) by JC‐1 staining. We found that the membrane potential in ageing oocytes after microinjection was significantly increased compared with that in control oocytes (0.32 ± 0.04 vs. 1.22 ± 0.13, *p* < 0.0001) (Figure 4E,F).

### 3.4 EnMSC mitochondrial transfer promoted preimplantation embryo development and increased birth rates after embryo transplantation

**FIGURE 5:**
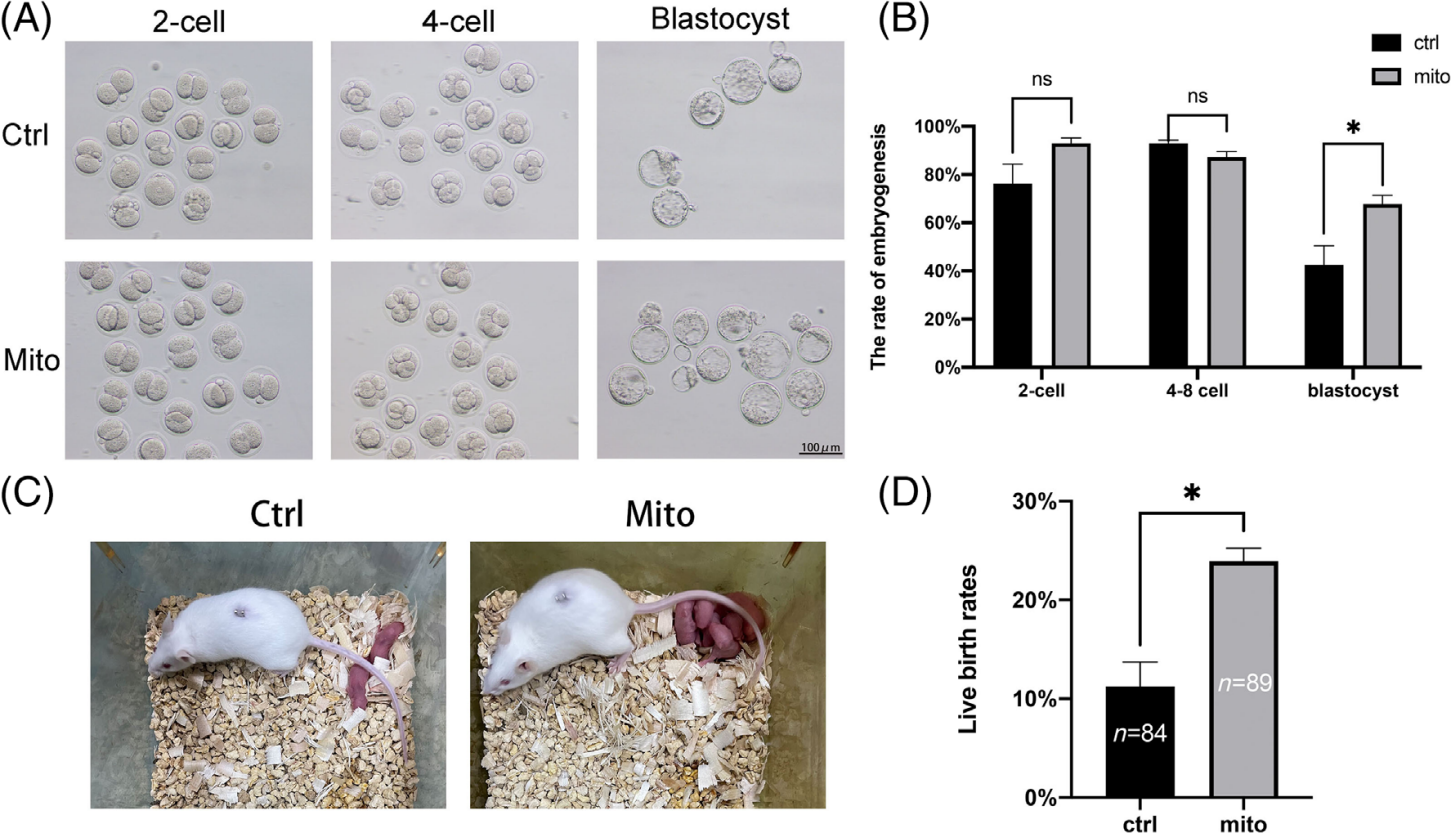
Embryogenesis was promoted and fertility was recovered in aged mice through endometrial mesenchymal stem cell (EnMSC) mitochondrial transfer. (A) Images of embryos cultured to different stages in the two groups. (B) Early embryogenesis was recorded. The group of aged mice subjected to mitochondrial transfer showed greater blastocyst rates than the control group. (C) Images of pups born after fallopian tube transplantation in the two groups. (D) The numbers of pups were calculated, and the results indicated that the live birth rate was increased in the mito‐group. The data are the mean ± SEM of three replicates; *n* shows the number of oocytes; and asterisk (*) indicates statistically significant differences (*p* < 0.05).

To further assess, the effects of mitochondrial supplementation on embryo development, EnMSC mitochondria microinjection combining with ICSI were carried out into MII oocytes, and control oocytes were injected with HTF. After 4 days of in vitro culture, the numbers of embryos that developed to the blastocyst stage significantly increased after mitochondrial injection (42.44% ± 8.02% vs. 67.72% ± 3.68%, *p* < 0.05) (Figure 5A,B, Table S1).

To test whether mitochondrial supplementation could improve in vivo embryo development in aged mice, embryo transplantation was performed after mitochondrial microinjection combined with ICSI. The results showed that the birth rate of oocytes with mitochondrial supplementation was significantly higher than that of the control oocytes (11.23% ± 2.48% vs. 23.95% ± 1.29%, *p* < 0.05) (Figure 5CD and Table S2).

## 4 DISCUSSION

Mitochondrial supplementation has been proposed as a promising therapeutic strategy to improve the competence and postfertilization development of aged oocytes. Early studies showed that healthy offspring were produced by cytoplasmic transfer from healthy oocytes into dysplastic oocytes. However, this approach resulted in potential late‐stage physical health problems due to mtDNA heteroplasmy.^27^, ^28^ To overcome this problem, OSCs and granulosa cells have been used as sources of mitochondria. However, the existence of OSCs is still controversial,^29^ and the copy number of mtDNA is difficult to calculate. Furthermore, the quantity and quality of extractable mitochondria from OSCs have made the technology somewhat questionable.^14^, ^15^ It has been reported that supplementation with mitochondria obtained from granulosa or cumulus cells can promote embryo development.^30^ Nevertheless, autologous granulosa cells are also affected by ageing, which increases the occurrence of mtDNA mutations and mitochondrial damage.^16^ In contrast, mitochondria derived from liver cells and mouse embryonic fibroblasts are unable to promote the development of mouse embryos. This failure is probably due to the cell‐specific nature of mitochondria. The mitochondria of somatic cells in other tissues do not function in oocytes because they are highly differentiated.^17^, ^31^

MSC‐based mitochondrial transfer has been used in regenerative medicine research in recent years, such as research on the treatment of respiratory system, kidney, cardiac and brain injury.^32^, ^33^, ^34^, ^35^, ^36^ There are several advantages of using MSCs as the sources of mitochondria for oocyte replenishment. First, compared with those of OSCs, the harvest and cultivation of mouse MSCs are more convenient and stable. Second, the morphology and metabolism of mitochondria in stem cells have been reported to be similar to those of mitochondria in MII oocytes.^37^, ^38^ Third, in this study, we found that EnMSCs have higher △Ψm and lower ROS levels than primary ESCs, which indicates that the mitochondrial function of EnMSCs is less susceptible to the effects of maternal ageing than that of somatic cells such as granulosa cells. Autologous adipose tissue‐derived stem cells (ADSCs) have also been used as sources of mitochondria for oocyte supplementation. Our previous study found that mitochondria of autologous ADSCs can increase the quality of aged oocytes, the rate of blastocyst formation (15% vs. 30%) and the rate of live births (2% vs. 15.7%).^22^

The concept of endometrial stem cells was first proposed in 1978.^39^ It has been reported that endometrial stem cells have a strong regenerative capacity and play important roles in the periodic proliferation and exfoliation of the endometrium. Human EnMSCs were first identified as clonogenic stromal cells isolated from both functional and basalis endometrium.^40^ Subsequent studies have demonstrated that these stromal cells express classic MSC markers and possess the potential to differentiate into multiple cell types.^41^, ^42^ Endometrial stem cells of mice have been found by a label‐retaining cell (LRC) approach to include epithelial LRCs and stromal LRCs.^43^ In this study, we established an approach for extracting and culturing EnMSCs from aged mice by modifying previously reported methods.^44^ We also demonstrated that EnMSCs from aged mice displayed higher mitochondrial membrane potential and lower ROS levels than primary ESCs. More importantly, supplementation with EnMSC‐derived mitochondria by microinjection significantly improved the quality of aged oocytes. In addition, we found that the rate of aneuploidy significantly decreased in aged oocytes after mitochondrial supplementation, suggesting that mitochondria are also involved in the meiosis of oocytes. Furthermore, our results showed that mitochondrial supplementation promoted embryo development and increased the live birth rate. It has been reported that the mtDNA of embryos begins to replicate until the blastocyst stage in most mammals. The oocyte mitochondrial pool, which represents 30% of oocytes, plays a crucial role in embryo development.^9^ Thus, mitochondria in oocytes must provide the preimplantation embryos with enough energy to promote embryo development. Therefore, we speculate that mitochondrial supplementation in oocytes can promote early embryo development since it increases the amount of ATP in the oocytes.

As mentioned above, the collection of somatic cells or adipose‐derived MSCs as sources of mitochondrial supplementation is an invasive process. It has been reported that human menstrual blood mesenchymal stem cells show good therapeutic potential in the field of regenerative medicine for procedures such as cardiac repair, liver repair and premature ovarian failure treatment.^45^ For clinical treatment, the cells can be obtained from the patient's own menstrual blood as the source of mitochondrial supplementation. A noninvasive procedure for cell extraction ensures safety, and the strategy can eliminate mtDNA heteroplasmy.

Moreover, many other strategies have been carried out to improve oocyte quality in addition to mitochondrial supplementation. Theaflavin 3,3'‐digallate (TF3) administered via gavage increases the mitochondrial membrane potential of oocytes and decreases the abnormal spindle rate of oocytes in aged mice.^46^ In one study, nine‐month‐old mice were injected subcutaneously with coenzyme Q10 to restore oocyte mitochondrial function and fertility during reproductive ageing.^12^ Astaxanthin has been used in in vitro culture to ameliorate the quality of postovulatory ageing pig oocytes.^47^ It has been reported that ovarian function can be improved by injecting human amniotic MSCs into the ovaries of naturally aged mice.^48^ All of the above studies treated MII oocytes, which means that the oocytes had completed the first meiotic division. In contrast, our study aimed to add mitochondria to GV oocytes that had not completed the first meiotic division.

In summary, our study demonstrates that the mitochondria of EnMSCs improve oocyte quality, embryo development and fertility in aged mice. Based on an evaluation of the safety, efficacy and stability of mitochondrial sources for clinical treatment, the findings of this work might provide a valuable strategy for patients with declining oocyte quality due to ageing.

## AUTHOR CONTRIBUTIONS

Yuan‐Qing Yao, Jian‐Xiu Hao, Qi Zhang designed the project. Qi Zhang, Jian‐Xiu Hao and Bo‐Wen Liu completed most of the experiments. Ying‐Chun Ouyang and Ming‐Zhe Dong provided technical support. Jia‐Ni Guo is responsible for assisting the work during the experiment. Qi Zhang analysed the data and wrote the manuscript with the help of Zhen‐Bo Wang. Zhen‐Bo Wang and Fei Gao contributed to the revision of the manuscript. Yuan‐Qing Yao participated in the final revision of the manuscript. All authors contributed to the study and approved the final version of the manuscript.

## FUNDING INFORMATION

This work was supported by the National Natural Science Foundation of China (81801529), the National High Technology Research and Development Program (863 program) of China (2015AA020402).

## CONFLICT OF INTEREST

The study's authors affirm that there are no financial or commercial ties that may be viewed as having a potential conflict of interest.

## Supporting information


**TABLE S1.** Decreased developmental arrests of embryos were found in aged mice through endometrial mesenchymal stem cell mitochondrial transfer.
**TABLE S2:** Endometrial mesenchymal stem cell mitochondria transfer contributed to fertility recovery in aged mice.
**FIGURE S1.**Negative staining results for vimentin and cytokeratin. Phosphate‐buffered saline was used to stain the samples instead of primary antibodies as a negative control, bar = 200 μm.

## Data Availability

The original contributions from this study can be found in the article/Supplementary Files. For further information, please contact the corresponding authors.

## References

[1] Qiao J , Wang ZB , Feng HL , et al. The root of reduced fertility in aged women and possible therapentic options: current status and future perspects. Mol Aspects Med. 2014;38:54‐85. doi:10.1016/j.mam.2013.06.001 23796757

[2] Mikwar M , MacFarlane AJ , Marchetti F . Mechanisms of oocyte aneuploidy associated with advanced maternal age. Mutat Res Rev Mutat Res. 2020;785:108320. doi:10.1016/j.mrrev.2020.108320 32800274

[3] Bebbere D , Coticchio G , Borini A , Ledda S . Oocyte aging: looking beyond chromosome segregation errors. J Assist Reprod Genet. 2022;39:793‐800. doi:10.1007/s10815-022-02441-z 35212880 PMC9051005

[4] Duncan FE , Jasti S , Paulson A , Kelsh JM , Fegley B , Gerton JL . Age‐associated dysregulation of protein metabolism in the mammalian oocyte. Aging Cell. 2017;16:1381‐1393. doi:10.1111/acel.12676 28994181 PMC5676066

[5] Yoldemir T . Fertility in midlife women. Climacteric. 2016;19:240‐246. doi:10.3109/13697137.2016.1164133 27098490

[6] Meldrum DR , Casper RF , Diez‐Juan A , Simon C , Domar AD , Frydman R . Aging and the environment affect gamete and embryo potential: can we intervene? Fertil Steril. 2016;105:548‐559. doi:10.1016/j.fertnstert.2016.01.013 26812244

[7] Kirillova A , Smitz JEJ , Sukhikh GT , Mazunin I . The role of mitochondria in oocyte maturation. Cell. 2021;10(9):2484. doi:10.3390/cells10092484 PMC8469615 34572133

[8] Boguenet M , Bouet PE , Spiers A , Reynier P , May‐Panloup P . Mitochondria: their role in spermatozoa and in male infertility. Hum Reprod Update. 2021;27:697‐719. doi:10.1093/humupd/dmab001 33555313

[9] May‐Panloup P , Boguenet M , Hachem HE , Bouet PE , Reynier P . Embryo and its mitochondria. Antioxidants (Basel). 2021;10(2):139. doi:10.3390/antiox10020139 33498182 PMC7908991

[10] Cozzolino M , Marin D , Sisti G . New Frontiers in IVF: mtDNA and autologous germline mitochondrial energy transfer. Reprod Biol Endocrinol. 2019;17:55. doi:10.1186/s12958-019-0501-z 31299996 PMC6626406

[11] Chiang JL , Shukla P , Pagidas K , et al. Mitochondria in ovarian aging and reproductive longevity. Ageing Res Rev. 2020;63:101168. doi:10.1016/j.arr.2020.101168 32896666 PMC9375691

[12] Ben‐Meir A , Burstein E , Borrego‐Alvarez A , et al. Coenzyme Q10 restores oocyte mitochondrial function and fertility during reproductive aging. Aging Cell. 2015;14:887‐895. doi:10.1111/acel.12368 26111777 PMC4568976

[13] Fragouli E , Spath K , Alfarawati S , et al. Altered levels of mitochondrial DNA are associated with female age, aneuploidy, and provide an independent measure of embryonic implantation potential. PLoS Genet. 2015;11:e1005241. doi:10.1371/journal.pgen.1005241 26039092 PMC4454688

[14] Gosden RG , Johnson MH . Can oocyte quality be augmented? Reprod Biomed Online. 2016;32:551‐555. doi:10.1016/j.rbmo.2016.04.001 27261842

[15] Labarta E , de los Santos MJ , Herraiz S , et al. Autologous mitochondrial transfer as a complementary technique to intracytoplasmic sperm injection to improve embryo quality in patients undergoing in vitro fertilization‐a randomized pilot study. Fertil Steril. 2019;111:86‐96. doi:10.1016/j.fertnstert.2018.09.023 30477915

[16] May‐Panloup P , Boucret L , Chao de la Barca JM , et al. Ovarian ageing: the role of mitochondria in oocytes and follicles. Hum Reprod Update. 2016;22:725‐743. doi:10.1093/humupd/dmw028 27562289

[17] Igarashi H , Takahashi T , Abe H , Nakano H , Nakajima O , Nagase S . Poor embryo development in post‐ovulatory in vivo‐aged mouse oocytes is associated with mitochondrial dysfunction, but mitochondrial transfer from somatic cells is not sufficient for rejuvenation. Hum Reprod. 2016;31:2331‐2338. doi:10.1093/humrep/dew203 27591230

[18] Ferreira AF , Soares M , Almeida Reis S , Ramalho‐Santos J , Sousa AP , Almeida‐Santos T . Does supplementation with mitochondria improve oocyte competence? A systematic review. Reproduction. 2021;161:269‐287. doi:10.1530/rep-20-0351 33275117

[19] Vasanthan J , Gurusamy N , Rajasingh S , et al. Role of human mesenchymal stem cells in regenerative therapy. Cell. 2020;10(1):54. doi:10.3390/cells10010054 PMC7823630 33396426

[20] He Y , Han Y , Ye Y . Therapeutic potential of menstrual blood‐derived stem cell transplantation for intrauterine adhesions. Front Surg. 2022;9:847213. doi:10.3389/fsurg.2022.847213 35274000 PMC8901573

[21] Wen M , Kwon Y , Wang Y , Mao JH , Wei G . Elevated expression of UBE2T exhibits oncogenic properties in human prostate cancer. Oncotarget. 2015;6:25226‐25239. doi:10.18632/oncotarget.4712 26308072 PMC4694827

[22] Wang ZB , Hao JX , Meng TG , et al. Transfer of autologous mitochondria from adipose tissue‐derived stem cells rescues oocyte quality and infertility in aged mice. Aging (Albany NY). 2017;9:2480‐2488. doi:10.18632/aging.101332 29283885 PMC5764387

[23] Huang L , Meng TG , Ma XS , et al. Rad9a is involved in chromatin decondensation and post‐zygotic embryo development in mice. Cell Death Differ. 2019;26:969‐980. doi:10.1038/s41418-018-0181-9 30154445 PMC6461798

[24] Cao L , Shitara H , Horii T , et al. The mitochondrial bottleneck occurs without reduction of mtDNA content in female mouse germ cells. Nat Genet. 2007;39:386‐390. doi:10.1038/ng1970 17293866

[25] Zhao BW , Sun SM , Xu K , et al. FBXO34 regulates the G2/M transition and anaphase entry in meiotic oocytes. Front Cell Dev Biol. 2021;9:647103. doi:10.3389/fcell.2021.647103 33842473 PMC8027338

[26] Dominici M , le Blanc K , Mueller I , et al. Minimal criteria for defining multipotent mesenchymal stromal cells. The International Society for Cellular Therapy position statement. Cytotherapy. 2006;8:315‐317. doi:10.1080/14653240600855905 16923606

[27] Barritt J , Willadsen S , Brenner C , Cohen J . Cytoplasmic transfer in assisted reproduction. Hum Reprod Update. 2001;7:428‐435. doi:10.1093/humupd/7.4.428 11476356

[28] van den Ameele J , Li AYZ , Ma H , Chinnery PF . Mitochondrial heteroplasmy beyond the oocyte bottleneck. Semin Cell Dev Biol. 2020;97:156‐166. doi:10.1016/j.semcdb.2019.10.001 31611080

[29] Hainaut M , Clarke HJ . Germ cells of the mammalian female: a limited or renewable resource?†. Biol Reprod. 2021;105:774‐788. doi:10.1093/biolre/ioab115 34114006 PMC8511662

[30] Hua S , Zhang Y , Li XC , et al. Effects of granulosa cell mitochondria transfer on the early development of bovine embryos in vitro. Cloning Stem Cells. 2007;9:237‐246. doi:10.1089/clo.2006.0020 17579556

[31] Zhang C , Tao L , Yue Y , et al. Mitochondrial transfer from induced pluripotent stem cells rescues developmental potential of in vitro fertilized embryos from aging femalesdagger. Biol Reprod. 2021;104:1114‐1125. doi:10.1093/biolre/ioab009 33511405

[32] Ding WX , Yin XM . Mitophagy: mechanisms, pathophysiological roles, and analysis. Biol Chem. 2012;393:547‐564. doi:10.1515/hsz-2012-0119 22944659 PMC3630798

[33] Islam MN , das SR , Emin MT , et al. Mitochondrial transfer from bone‐marrow‐derived stromal cells to pulmonary alveoli protects against acute lung injury. Nat Med. 2012;18:759‐765. doi:10.1038/nm.2736 22504485 PMC3727429

[34] Vallabhaneni KC , Haller H , Dumler I . Vascular smooth muscle cells initiate proliferation of mesenchymal stem cells by mitochondrial transfer via tunneling nanotubes. Stem Cells Dev. 2012;21:3104‐3113. doi:10.1089/scd.2011.0691 22676452 PMC3495124

[35] Plotnikov EY , Khryapenkova TG , Galkina SI , Sukhikh GT , Zorov DB . Cytoplasm and organelle transfer between mesenchymal multipotent stromal cells and renal tubular cells in co‐culture. Exp Cell Res. 2010;316:2447‐2455. doi:10.1016/j.yexcr.2010.06.009 20599955

[36] Babenko VA , Silachev DN , Zorova LD , et al. Improving the post‐stroke therapeutic potency of mesenchymal multipotent stromal cells by cocultivation with cortical neurons: the role of crosstalk between cells. Stem Cells Transl Med. 2015;4:1011‐1020. doi:10.5966/sctm.2015-0010 26160961 PMC4542870

[37] Schatten H , Sun QY , Prather R . The impact of mitochondrial function/dysfunction on IVF and new treatment possibilities for infertility. Reprod Biol Endocrinol. 2014;12:111. doi:10.1186/1477-7827-12-111 25421171 PMC4297407

[38] Li Q , Gao Z , Chen Y , Guan MX . The role of mitochondria in osteogenic, adipogenic and chondrogenic differentiation of mesenchymal stem cells. Protein Cell. 2017;8:439‐445. doi:10.1007/s13238-017-0385-7 28271444 PMC5445026

[39] Prianishnikov VA . On the concept of stem cell and a model of functional‐morphological structure of the endometrium. Contraception. 1978;18:213‐223. doi:10.1016/s0010-7824(78)80015-8 569035

[40] Chan RW , Schwab KE , Gargett CE . Clonogenicity of human endometrial epithelial and stromal cells. Biol Reprod. 2004;70:1738‐1750. doi:10.1095/biolreprod.103.024109 14766732

[41] Gargett CE , Schwab KE , Zillwood RM , Nguyen HP , Wu D . Isolation and culture of epithelial progenitors and mesenchymal stem cells from human endometrium. Biol Reprod. 2009;80:1136‐1145. doi:10.1095/biolreprod.108.075226 19228591 PMC2849811

[42] Rajaraman G , White J , Tan KS , et al. Optimization and scale‐up culture of human endometrial multipotent mesenchymal stromal cells: potential for clinical application. Tissue Eng Part C Methods. 2013;19:80‐92. doi:10.1089/ten.TEC.2011.0718 22738377 PMC3522126

[43] Chan RW , Gargett CE . Identification of label‐retaining cells in mouse endometrium. Stem Cells. 2006;24:1529‐1538. doi:10.1634/stemcells.2005-0411 16456137

[44] De Clercq K , Hennes A , Vriens J . Isolation of mouse endometrial epithelial and stromal cells for In vitro decidualization. J Vis Exp. 2017;(121):55168. doi:10.3791/55168 28287563 PMC5408775

[45] Bozorgmehr M , Gurung S , Darzi S , et al. Endometrial and menstrual blood mesenchymal stem/stromal cells: biological properties and clinical application. Front Cell Dev Biol. 2020;8:497. doi:10.3389/fcell.2020.00497 32742977 PMC7364758

[46] He J , Yao G , He Q , et al. Theaflavin 3, 3'‐digallate delays ovarian aging by improving oocyte quality and regulating granulosa cell function. Oxid Med Cell Longev. 2021;2021:7064179. doi:10.1155/2021/7064179 34925699 PMC8674650

[47] Jia BY , Xiang DC , Shao QY , et al. Inhibitory effects of astaxanthin on postovulatory porcine oocyte aging in vitro. Sci Rep. 2020;10:20217. doi:10.1038/s41598-020-77359-6 33214659 PMC7677382

[48] Ding C , Zou Q , Wang F , et al. Human amniotic mesenchymal stem cells improve ovarian function in natural aging through secreting hepatocyte growth factor and epidermal growth factor. Stem Cell Res Ther. 2018;9:55. doi:10.1186/s13287-018-0781-9 29523193 PMC5845161

