# Supplementary material for: Supplementation of mitochondria from endometrial mesenchymal stem cells improves oocyte quality in aged mice
Source: Cell Prolif. 2022 Dec 8;56(3):e13372. doi: 10.1111/cpr.13372 (PMC9977672; doi:10.1111/cpr.13372)

Table S1. Decreased developmental arrests of embryos were found in aging mice through EnMSCs mitochondria transfer.

|  | Zygotes | 2-cell | **Developmental arrests rates (%)** | 4-8-cell | **Developmental arrests rates (%)** | Blastocyst | **Developmental arrests rates(%)** |
| --- | --- | --- | --- | --- | --- | --- | --- |
| Ctrl 1 | 33 | 23 | 30.30 | 22 | 13.04 | 6 | 72.72 |
| Ctrl 2 | 13 | 12 | 7.69 | 11 | 8.33 | 6 | 45.45 |
| Ctrl 3 | 18 | 12 | 33.33 | 11 | 8.33 | 5 | 54.55 |
| Mean ± SEM |  |  | 23.77±8.09 |  | 9.9±1.57 |  | 57.57±8.02 |
| Mito 1 | 26 | 23 | 11.54 | 19 | 17.39 | 12 | 36.84 |
| Mito 2 | 23 | 22 | 4.35 | 20 | 9.09 | 13 | 35 |
| Mito 3 | 19 | 18 | 5.26 | 16 | 11.11 | 12 | 25 |
| Mean ± SEM |  |  | 7.05±2.26 |  | 12.53±2.50 |  | 32.28±3.68 |

Table S2: EnMSCs mitochondria transfer contributed to fertility recovery in aging mice.

|  | Control group | | | Mito group | | |
| --- | --- | --- | --- | --- | --- | --- |
|  | Embryos(n) | Pups | **Live birth rates (%)** | Embryos(n) | Pups | **Live birth rates (%)** |
| First transplant | 20 | 2 | 10 | 29 | 7 | 24.14 |
| Second transplant | 25 | 4 | 16 | 37 | 8 | 21.62 |
| Third transplant | 39 | 3 | 7.69 | 23 | 6 | 26.09 |
| Sum/Average ratio | 84 | 9 | 11.23 | 89 | 21 | 23.95 |

Figure S1．Negative staining results for vimentin and cytokeratin. PBS was used to stain the samples instead of primary antibodies as negative control, bar=200μm


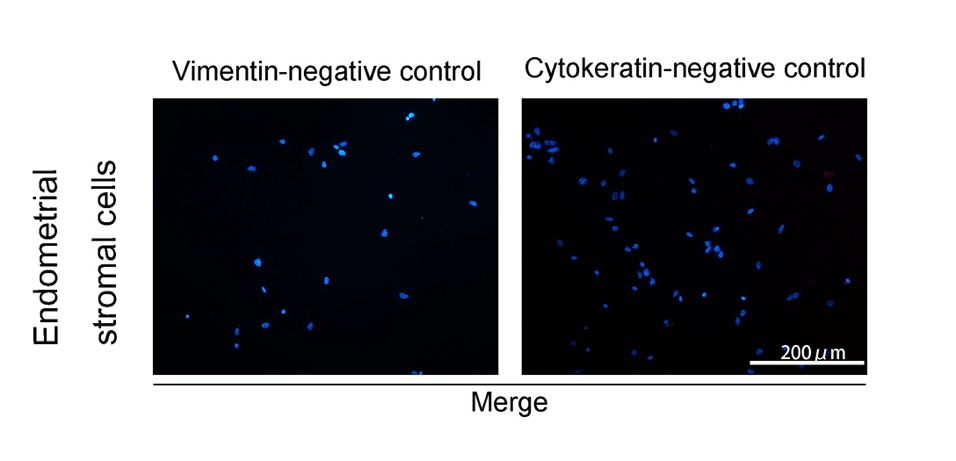

Supplement: Supplementary file 1 — TABLE S1. Decreased developmental arrests of embryos were found in aged mice through endometrial mesenchymal stem cell mitochondrial transfer. TABLE S2: Endometrial mesenchymal stem cell mitochondria transfer contributed to fertility recovery in aged mice. FIGURE S1.Negative staining results for vimentin and cytokeratin. Phosphate‐buffered saline was used to stain the samples instead of primary antibodies as a negative control, bar = 200 μm. [file CPR-56-e13372-s001.docx]
